# From Oxidative Stress to Ageing via Lifestyle, Nutraceuticals, Polypharmacy, and Neuropsychological Factors

**DOI:** 10.1155/2018/6352689

**Published:** 2018-09-30

**Authors:** Ilaria Peluso, Maura Palmery, Nagendra Sastry Yarla, George Perry, Mohammad A. Kamal

**Affiliations:** ^1^Research Center for Food and Nutrition, Council for Agricultural Research and Economics (CREA-AN), Rome, Italy; ^2^Department of Physiology and Pharmacology “V. Erspamer”, Sapienza University of Rome, Italy; ^3^Department of Animal Biology, School of Life Sciences, University of Hyderabad, Gachibowli, Hyderabad 500 046, Telangana, India; ^4^College of Science, University of Texas at San Antonio, TX, USA; ^5^King Fahd Medical Research Center, King Abdulaziz University, Jeddah 21589, Saudi Arabia; ^6^Enzymoics, 7 Peterlee Place, Hebersham, NSW 2770, Australia; ^7^Novel Global Community Educational Foundation, Australia

Lifestyle factors including dietary habit, physical activity, smoke, and physiological stress are modifiable determinants of the oxidative stress and the chronic low-grade inflammation involved in the pathogenesis and progression of many ageing-associated diseases. In a cross-sectional pilot study, Seyedsadjadi et al. found relationships between a comprehensive redox balance lifestyle score (RBLS) and carotid intima-media thickness and total antioxidant capacity (TAC)/hydroperoxide (HPX) ratio. The RBLS included visceral adipose tissue, nutritional factors (polyunsaturated fatty acids, transfatty acids, iron, vitamin E, vitamin C, and carotenoids intakes), caffeine and alcohol intakes, smoking history, physical activity, depression scores (stress and anxiety), Pittsburgh sleep quality index, and apnoea risk. Moreover, RBLS was related with the inflammatory markers C-reactive protein and interleukin-1*β*. In this context, Ma et al. reported that melatonin suppressed the activation of the prolonged nucleotide-binding domain and leucine-rich repeat pyrin domain-containing 3 (NLRP3) inflammasome and the secretion of IL-1*β* within the atherosclerotic lesions, in the atherosclerotic model high-fat-diet ApoE^−/−^ mice. Furthermore, in this study, melatonin induced sirtuin (SIRT)3 activation. On the other hand, two studies in the present special issue investigated the modulation of SIRT1 by nutraceuticals. Da Pozzo et al. found increases in SIRT1, nuclear factor erythroid 2-related factor 2 (NRF2), forkhead/winged helix box gene, and group O (FOXO)3 mRNA levels in the hearts of old mice fed with lyophilized bergamot juice (1 mg/kg/day) diluted in water for three months. Corbi et al. investigated the effects of treatment (80 days) in rabbits with various supplements including *Lippia citriodora* extract, containing verbascoside 5 mg/kg feed (VB), 350 mg of root extract of *Raphanus sativus*/kg feed (RAP), or 5 mg of lycopene, extracted from tomato fruit/kg feed (LYC). SIRT1 activity was higher in the rabbit's heart and liver of animals treated with VB, compared to the groups receiving control, RAP, and LYC. However, no differences were found in SIRT1 protein expression between groups in both the heart and liver of the rabbits. On the other hand, treatment with VB, RAP, and LYC resulted in a marked improvement in the blood lipid and glycaemic profile in respect to control and all three plant extracts induced a significant reduction in oxidant parameters as well as an increase in antioxidant tissue activity and vitamin A and E levels.

Ageing per se is associated with a decline in the antioxidant defenses, with telomere-shortening processes, with immunosenescence and with mitochondrial dysfunction. In a meta-analysis of animal studies, Braakhuis et al. reported decreased nitrotyrosine concentration (190 animals, SMD −0.67, 95% CI (−1.30, −0.05), *p* = 0.04), protein carbonyl concentration (182 animals, SMD −0.13, 95% CI (−0.44, 0.18), *p* = 0.41), and increase in the membrane potential (63 animals, MD 11.44, 95% CI (1.28–21.60), *p* = 0 03) after treatment with the mitochondrion-targeted antioxidant MitoQ.

Although diet and exercise are the major players in the prevention of frailty in the elderly, moods can affect both the antioxidant defensive and the immune systems. Being the activation of hypothalamus-pituitary-adrenocortical (HPA) axis is crucial in stress response and the bed nucleus of the stria terminalis (BST), a mediator of the HPA axis responses to stress; Karnia et al. investigated the effect of the electrical stimulation (four weeks) of BTS. BTS caused oxidative stress (i.e., higher level of lipid peroxidation markers, lower level of protein oxidation marker, and elevated antioxidant enzyme activity) in skeletal muscle of rats. Besides, increased plasma corticosterone concentration was found. From that, the authors suggested that these findings could have also potential implication showing that reaction to the long-term “psychological stress” may lead to free radical damage of muscle.

Controversies surrounding the usefulness of nutraceuticals in elderlies exist, and possible food-drug interaction in aged people is an underlooked aspect of geriatric patients that could affect metabolic pathways. The human study (Fodor et al.), included in this special issue, reported that resveratrol (100 and 200 mg for 12 months) improved blood pressure, body mass index, glucose (in nondiabetic patients), and lipid profile, in patients who had a stroke in the last 12 months and who underwent allopathic (medical) treatment combined with medical physical rehabilitation, whereas no adverse effects were observed.

Behavioural, epigenetic (dietary phytochemicals, drugs), and lifestyle (dietary intake, physical activity, and smoking habit) factors must be taken into account in the management of geriatric patients in order to assess individual risk or benefit of dietary and/or physical activity advice in the context of a personalized drug therapy. In this context, spatiotemporal distribution of drug is particularly relevant. Kalangi et al. tested in the carrageenan-induced mouse paw edema model of inflammation the hypothesis that nanotechnology in combination with optical imaging using quantum dot- (QD-) tagged biological macromolecules can be used to estimate the spatiotemporal distribution of the anti-inflammatory Celecoxib. *In vivo* imaging of QD-Celecoxib conjugates showed clear localization in the inflamed tissue of mouse paw within 3 h, with a gradual increase reaching a maximum and a later decline followed by an increase in urinary bladder region.

In conclusion, we sincerely hope that this special issue contributes to add knowledge in age-related disease management. However, more studies are needed in order to shed light on the relationship between neuropsychological factors and oxidative stress, as well as on nongenetic or genetic influences that affect immune, anti-inflammatory, and antioxidant aspects related to accelerated ageing.

